# Magnetic Resonance‐Guided Focused Ultrasound Thalamotomy for Focal Hand Dystonia: A Pilot Study

**DOI:** 10.1002/mds.28613

**Published:** 2021-05-29

**Authors:** Shiro Horisawa, Toshio Yamaguchi, Keiichi Abe, Hiroki Hori, Atsushi Fukui, Mutsumi Iijima, Masatake Sumi, Kenichi Hodotsuka, Yoshiyuki Konishi, Takakazu Kawamata, Takaomi Taira

**Affiliations:** ^1^ Department of Neurosurgery Neurological Institute, Tokyo Women's Medical University Tokyo Japan; ^2^ Department of Radiology Shinyurigaoka General Hospital Kanagawa Japan; ^3^ Department of Neurology Neurological Institute, Tokyo Women's Medical University Tokyo Japan; ^4^ Faculty of Advanced Techno‐Surgery Institute of Advanced Biomedical Engineering & Science, Graduate School of Medicine Tokyo Japan

**Keywords:** magnetic resonance‐guided focused ultrasound thalamotomy, focal hand dystonia, ventro‐oral nucleus

## Abstract

**Background:**

The efficacy of magnetic resonance‐guided focused ultrasound (MRgFUS) thalamotomy for the treatment of focal hand dystonia (FHD) is not well known.

**Objective:**

We aimed to prospectively investigate the efficacy of MRgFUS thalamotomy for the treatment of FHD.

**Methods:**

We performed MRgFUS thalamotomy of the ventro‐oral (Vo) nucleus in 10 patients with FHD. We evaluated the scores of the Writer's Cramp Rating Scale (WCRS, 0–30; higher scores indicating greater severity), Tubiana Musician's Dystonia Scale (TMDS, 0–5; lower scores indicating greater severity), and Arm Dystonia Disability Scale (ADDS, 0%–100%; lower scores indicating greater disability) at baseline and 3 and 12 months post‐treatment.

**Results:**

WCRS, TMDS, and ADDS scores significantly improved from 6.3 ± 2.7, 1.4 ± 0.5, and 58.7% ± 14.3% at baseline to 1.6 ± 3.1 (*P* = 0.011), 5.0 ± 0 (*P* = 0.0001), and 81.6% ± 22.9% (*P* = 0.0229) at 12 months, respectively. There was one prolonged case of dysarthria at 12 months.

**Conclusion:**

We show that MRgFUS Vo‐thalamotomy significantly improved FHD. © 2021 The Authors. *Movement Disorders* published by Wiley Periodicals LLC on behalf of International Parkinson and Movement Disorder Society

## 1 Introduction

Focal hand dystonia (FHD) is an idiopathic, adult‐onset disorder in most cases and manifests only while performing specific tasks.^1^ Writer's cramp and musician's dystonia are the most well‐known types of FHD, both of which cause dystonic muscle contractions in hand muscles only while writing or playing musical instruments. The prevalence of this condition is 1.2–1.5 per 100,000 persons.^2^, ^3^, ^4^ In some specific populations, such as professional musicians or athletes, the prevalence is much higher than in the general population; approximately 1%–2% of professional musicians are affected by FHD.^5^, ^6^

The ventro‐oral (Vo) nucleus is one of the main output terminators from the basal ganglia, and lesioning or stimulation of this nucleus has been reported to improve FHD.^7^, ^8^ Magnetic resonance (MR)‐guided focused ultrasound (MRgFUS) thalamotomy, which allows intracranial focal lesioning without an incision, has been reported to be an effective and less invasive procedure for the treatment of tremor and Parkinson's disease.^9^, ^10^ MRgFUS thalamotomy produces thermal lesions, similarly to radiofrequency thalamotomy, and is expected to have similar effects to radiofrequency Vo‐thalamotomy on FHD. However, its efficacy for the treatment of FHD has been investigated in only two patients thus far.^11^, ^12^ Therefore, we prospectively investigated the efficacy of MRgFUS Vo‐thalamotomy in 10 patients with FHD.

## 2 Patients and Methods

### 2.1 Study Design

This was a prospective, open‐label, non‐controlled pilot study performed at the Tokyo Women's Medical University, Shinjuku, Tokyo, Japan, except for the FUS procedures, which were conducted at the Shin‐Yurigaoka General Hospital, Kawasaki, Yokohama, Japan. The protocol was reviewed and approved by the University Hospital Medical Information Network Center and the Ethics Committees of both participating hospitals. All patients provided written informed consent before inclusion in this study.

### 2.2 Patients

From April 2017 through May 2018, we enrolled 10 patients with FHD. Severe FHD was defined as a score of <3 on the Tubiana Musician's Dystonia Scale (TMDS) or > 8 on the Writer's Cramp Rating Scale (WCRS). The diagnosis of FHD was confirmed by a neurologist or a neurosurgeon specializing in movement disorders (T.T., S.H., M.I.). The exclusion criteria included the following: history of stereotactic surgery or cerebral stroke, diagnosis of an unstable cardiac or psychiatric disease, coagulopathy, skull density ratio of ≤0.3, and cognitive impairment (a score of >24 [out of 30] on the Mini‐Mental State Examination).

### 2.3 Evaluations

The primary clinical endpoint was defined as a change in the WCRS score (0–30, with higher scores indicating greater severity) in writer's cramp or the TMDS score (1–5, with lower scores indicating greater severity) in musician's dystonia. The secondary clinical endpoint was the change in the score of the Arm Dystonia Disability Scale (ADDS; 0%–100%, with lower scores indicating greater disability).^13^ Patients were assessed for these clinical endpoints and adverse events at baseline, 1 week, 1 month, 3 months, 6 months, and 12 months after treatment. Each assessment was videotaped. Adverse events were classified in relation to thalamotomy, stereotactic frame, sonication, and unrelated. Transient adverse events were defined as those resolving by the 12‐month follow‐up. Serious adverse events were those requiring hospital admission or prolonged hospitalization. Magnetic resonance imaging (MRI) was performed at baseline, 3 months, and 12 months after treatment.

### 2.4 FUS Procedure

The stereotactic coordinates of the Vo nucleus were 1.0–1.5 mm posterior, 14–15 mm lateral, and 1.5–2.5 mm superior to the mid‐commissural point, which corresponds to the junction between the ventro‐oral anterior and posterior of the thalamus according to the stereotactic atlas of Schaltenbrand and Wahren.^14^ The procedure was performed with 3‐T MRI (GE Healthcare Technologies, Waukesha, WI) and ExAblate Neuro systems (Insightec, Tirat Carmel, Israel). At least two sonications above 55°C were administered to the target. Clinical assessment for dystonic symptoms and adverse events was performed after each sonication. After the procedure, the stereotactic frame was removed and the patients were discharged the following day.

### 2.5 Statistical Analysis

The data did not follow a normal distribution. Friedman's test was used to determine the primary (change in TMDS and WCRS scores) and secondary clinical endpoints (change in ADDS score) across six study visits: at baseline, 1 week, 1 month, 3 months, 6 months, and 12 months after treatment. Wilcoxon's signed rank test was used to compare the primary and secondary endpoints between baseline and 12 months. Bonferroni's correction was used for multiple comparisons. Statistical analysis was performed using the JMP statistical package (version 13.0.0; SAS Institute, Cary, NC). All statistical tests were two tailed, and *P* < 0.05 was considered to be statistically significant.

## 3 Results

### 3.1 Patients

**FIG. 1:**
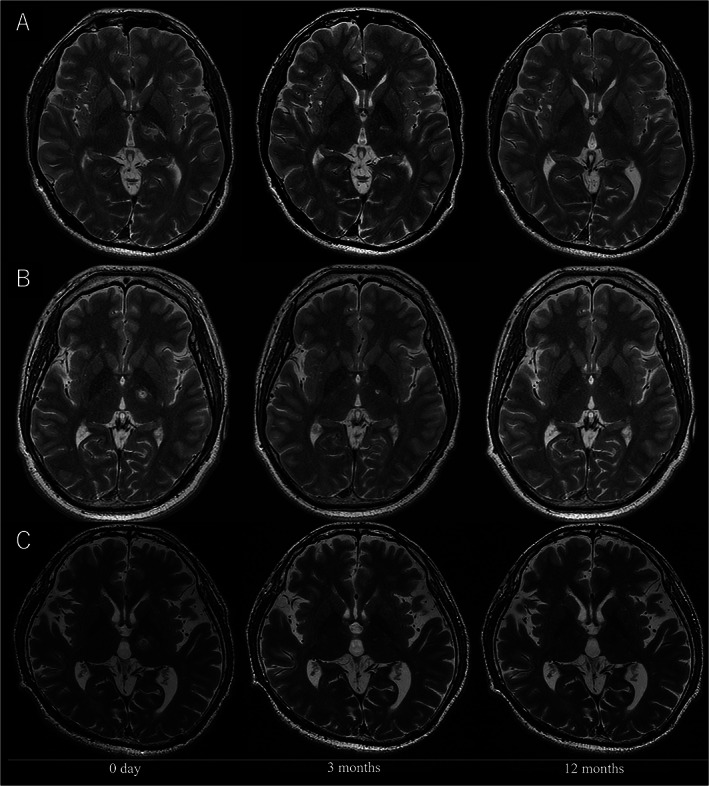
Representative magnetic resonance images obtained before and after treatment. The lesion unexpectedly encroached on the posterior limb of the internal capsule (case 4: **A**, case 7: **B**). Case 9 (**C**) showing the precise lesion on the intended target.

We enrolled 10 patients with severe FHD: five with musician's dystonia (four were professional players), three with writer's cramp, and two patients with professional dart‐related FHD. All five patients with musician's dystonia and one patient with dart‐related FHD also manifested writer's cramp. One patient had isolated dart‐related FHD. This patient's dystonic symptoms were severe; thus, we decided to enroll this patient in this study. The patients' baseline characteristics are shown in Table SS1. Representative postoperative MRI scans are shown in Figure 1.

### 3.2 Clinical Effects of MRgFUS

**FIG. 2:**
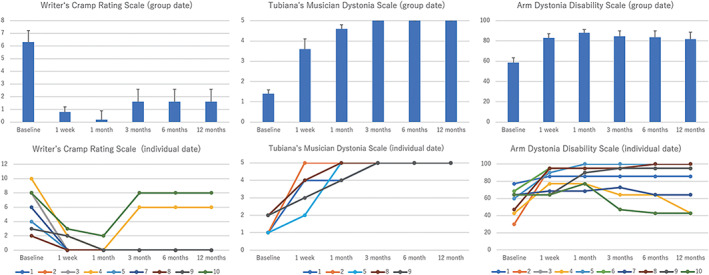
Changes in primary and secondary clinical endpoints during the study period. Primary clinical endpoints were the scores of the Writer's Cramp Rating Scale (WCRS; ranging from 0 to 30, with higher scores indicating greater severity) and Tubiana Musician's Dystonia Scale (TMDS; ranging from 1 to 5, with lower scores indicating greater severity), which evaluate dystonia severity. Secondary clinical endpoint was the score of the Arm Dystonia Disability Scale (ADDS; ranging from 0% to 100%, with lower scores indicating greater disability) to evaluate dystonia disability. Significant improvements were observed in the WCRS, TMDS, and ADDS scores throughout the study period in group data (upper row). Individual data are shown in the lower row.

The group and individual scores of each clinical assessment are described in Table SS1 and Figure 2. In nine patients with writer's cramp, the WCRS score was reduced by 71.4% (range: 0%–100%) at 12 months (6.1 ± 2.9 at baseline vs. 1.8 ± 3.3 at 12 months, *P* = 0.011). In five patients with musician's dystonia, the TMDS score was significantly improved from 1.4 ± 0.5 (range: 1–2) at baseline to 5.0 ± 0 (range: 5) at 12 months (*P* < 0.0001). All patients were evaluated by the ADDS, and the score significantly improved from 58.7% ± 14.3% (range: 30.0%–77.1%) at baseline to 81.6% ± 22.9% (range: 42.9%–100%) at 12 months (*P* = 0.025). Significant improvements were identified in the WCRS (*P* < 0.001), TMDS (*P* = 0.001), and ADDS (*P* = 0.001) scores. Five patients (cases 2, 6, 7, 8, and 9) were not working at baseline due to the symptoms, and four (cases 2, 6, 8, and 9) of them had returned to work by the 12‐month follow‐up. One patient (case 7) remained unemployed with poor treatment results. The clinical course of representative cases in writer's cramp (case 3) and musician's dystonia (case 2) are shown in videos S1 and S2. Symptom recurrence was noted in three patients (cases 4, 7, and 10), including the two with facial palsy, at the 3‐month follow‐up. The summary of the sonication can be found in Table S2.

### 3.3 Adverse Events

All adverse events related to thalamotomy, stereotactic frame, and sonication were transient (Table SS3). There was one serious adverse event, a suicide attempt by drug overdose, which was not related to thalamotomy, stereotactic frame, and sonication. The patient recovered without any deficit after a 5‐day hospitalization (case 7). The suicide attempt was made 2 months after the treatment, which led to the discovery that he intentionally concealed the history of repetitive suicide attempts and depression before enrollment in this trial. Three patients showed transient dysarthria (cases 5, 7, and 10), and dysarthria persisted at 12 months in one patient (case 4). Two patients had transient facial palsy. In both cases (cases 4 and 7), facial palsy developed by accidental lesion encroachment on the internal capsule (Fig. 1A,B) and resolved spontaneously within 3 months.

## 4 Discussion

This open‐label pilot study of 10 patients showed that unilateral MRgFUS Vo‐thalamotomy significantly improved the symptoms of FHD, as measured by the WCRS, TMDS, and ADDS. No serious adverse events were observed, except for the suicide attempt in one patient. The only adverse event at 12 months was mild dysarthria in one patient.

Thalamotomy using FUS has been reported mainly in the treatment of tremors associated with essential tremor and Parkinson's disease.^9^, ^10^, ^15^ Only two patients with FHD have been reported to be treated by FUS thalamotomy.^11^, ^12^ We have already reported several studies describing the efficacy of Vo‐thalamotomy using radiofrequency and gamma knife.^16^, ^17^, ^18^, ^19^, ^20^ In this study, the WCRS, TMDS, and ADDS scores significantly improved at 12 months compared to those at baseline. In our previous study investigating the results of radiofrequency Vo‐thalamotomy in 171 patients, the task‐specific FHD scores (1–5, with lower scores indicating greater severity), a scale that is almost the same as TMDS, before surgery and at the last available follow‐up (mean 47.36 months) were 1.72 ± 0.57 and 4.39 ± 1.07, respectively, which are similar to the results of the present study.^20^ In a limited number of cases, Vo‐deep brain  stimulation is also reported to be effective for hand dystonia.^21^, ^22^

In this study, three patients experienced recurrence of the dystonic symptoms at 3 months after treatment. In radiofrequency Vo‐thalamotomy, recurrence can also develop within 3 months after surgery in about 10% of patients with FHD, which results from insufficient or incorrect ablation over the entire Vo nucleus.^20^ Until the edema surrounding the thermocoagulation, which covers the entire Vo nucleus, disappears, symptomatic improvements may persist. The postoperative MRI scans of patients with recurring symptoms showed that the lesion was situated laterally from where we intended, suggesting that misalignment of the target was a possible cause of the recurrence. Compared to the MRI scan on the day of the treatment in the successful case 9 (Fig. 1B), the recurrent case 4 (Fig. 1A) showed that the volume of the intrathalamic lesion volume was small. Lesion encroachment on the internal capsule (cases 4, 7, and 10) may be related to poor outcomes and adverse events. In the time course of ADDS, cases 4 and 7 showed at 12 months a score similar to that at baseline after initial improvement. Case 10 showed a worsening score at 12 months than that at baseline due to disease progression. Case 7 made a suicide attempt by drug overdose 2 months after the treatment. Whether the Vo nucleus lesion had an impact on his psychological deterioration is uncertain. However, the unsatisfactory result may have attributed to the worsening of his psychological condition, ultimately leading to the suicide attempt.

Despite the limitations, including a short follow‐up period and small sample size, this pilot study suggests that FUS Vo‐thalamotomy may be an alternative treatment option for patients with FHD. A randomized controlled study with a larger sample size and a longer follow‐up period is warranted to elucidate the efficacy and safety of MRgFUS Vo‐thalamotomy for FHD.

## Author Roles

(1) Research Project: A. Conception, B. Organization, C. Execution; (2) Statistical Analysis: A. Design, B. Execution, C. Review and Critique; (3) Manuscript Preparation: A. Writing of the First Draft, B. Review and Critique.

S.H.: 1A, 1B, 1C, 2A, 2C, 3A, 3B

T.Y.: 1B, 1C

K.A.: 1B, 1C

H.H.: 1C

A.F.: 2B, 2C

M.I.: 1C

M.S.: 1C

K.H.: 1C

Y.K.: 1C

T.K.: 1B

T.T.: 1A, 1B, 1C, 2A, 3B

## Financial Disclosures (for the preceding 12 months)

This study was supported by a research grant from the Focused Ultrasound Foundation. The authors report no other funding sources or conflicts of interest in the previous 12 months, regardless of the relationship to the current research presented in this manuscript.

## Supporting information

**Video S1.** Pre‐ and post‐treatment conditions in musician's dystonia (professional pianist, patient #2)

**Video S2.** Pre‐ and post‐treatment conditions in writer's cramp (patient #3)

**Table S1.** Patient characteristics and clinical outcomes

**Table S2.** Summary of focused ultrasound procedures

**Table S3.** Adverse events
