# Supplementary material for: Magnetic Resonance‐Guided Focused Ultrasound Thalamotomy for Focal Hand Dystonia: A Pilot Study
Source: Mov Disord. 2021 May 29;36(8):1955–9. doi: 10.1002/mds.28613 (PMC8453941; doi:10.1002/mds.28613)
Supplement: Supplementary file 3 — Table S1. Patient characteristics and clinical outcomes [file MDS-36-1955-s004.docx]

Supplementary Table.1 Patient characteristics and clinical outcomes

| Case | Gender | Age at surgery (yr) | Age at onset (yr) | Duration of FHD (yr) | Handedness | Symptoms | Initial Affected tasks | Other affected tasks | WCRS/TMDS/ADDS | | | | | | Adverse events※ | | Recurrence | Lesion | | | |
| --- | --- | --- | --- | --- | --- | --- | --- | --- | --- | --- | --- | --- | --- | --- | --- | --- | --- | --- | --- | --- | --- |
|  |  |  |  |  |  |  |  |  |  |  |  |  |  |  |  |  |  | Location (mm) † | | | Volume (mm³)‡ |
|  |  |  |  |  |  |  |  |  | Baseline | 1 week | 1 month | 3 months | 6 months | 12 months | transient | prolonged |  | AP | LR | SI |  |
| 1 | M | 35 | 26 | 9 | R | Rt 2/3/4 finger flexion | Guitar | writing | 8/1/77.1 | 0/4/85.7 | 0/4/85.7 | 0/5/85.7 | 0/5/85.7 | 0/5/85.7 |  |  | No | 10.9 | 13.6 | 2.4 | 282 |
| 2 | F | 45 | 17 | 28 | R | Rt 4/5 flexion | Piano | writing | 8/1/30 | 0/5/95 | 0/5/95 | 0/5/95 | 0/5/95 | 0/5/95 |  |  | No | 10.3 | 13.8 | 3 | 324 |
| 3 | M | 58 | 37 | 21 | L | Rt wrist extension | Writing |  | 8/-/68.6 | 0/-/95 | 0/-/95 | 0/-/95 | 0/-/95 | 0/-/95 | unsteady gait |  | No | 11.1 | 14.4 | 2.9 | 226 |
| 4 | M | 45 | 43 | 2 | R | Rt wrist flexion, Rt 2-4 finger flexion | Writing |  | 10/-/42.9 | 2/-/77.1 | 0/-/77.14 | 6/-/64.3 | 6/-/64.3 | 6/-/42.9 | heifacial palsy | dysarthria | Yes | 11 | 14.9 | 0.5 | 392 |
| 5 | M | 34 | 20 | 14 | R | Lt 3rd finger flexion | Guitar | writing | 4/1/60 | 0/2/90 | 0/5/100 | 0/5/100 | 0/5/100 | 0/5/100 | dysarthria |  | No | 10.6 | 15.1 | 2 | 442 |
| 6 | M | 45 | 42 | 3 | R | Rt 1st finger flexion | Darts |  | -/-/68.6 | -/-/95 | -/-/95 | -/-/95 | -/-/95 | -/-/95 |  |  | No | 11 | 15.5 | 3.1 | 155 |
| 7 | M | 35 | 34 | 1 | R | Rt 2nd finger extension, Rt 1st finger stiffness | Darts | writing | 6/-/64.3 | 0/-/68.6 | 0/-/68.6 | 0/-/72.9 | 0/-/64.3 | 0/-/64.3 | dysarthria, unsteady gait |  | Yes | 10.3 | 15.5 | 2.1 | 255 |
| 8 | M | 52 | 47 | 5 | R | Rt 3/4/5 flexion | Guitar | writing | 2/2/47.1 | 0/4/95 | 0/5/95 | 0/5/95 | 0/5/100 | 0/5/100 |  |  | No | 11.2 | 15.6 | 2.7 | 286 |
| 9 | M | 56 | 51 | 5 | R | Rt wrist flexion | Japanese drums | writing | 3/2/64.3 | 2/3/64.3 | 0/5/90 | 0/5/95 | 0/5/95 | 0/5/95 |  |  | No | 9.9 | 15 | 2.3 | 661 |
| 10 | M | 27 | 17 | 10 | R | Right wrist stiffness, Right hand fingers flexion | Writing | Guitar | 8/-/64.3 | 3/-/64.3 | 2/-/77.1 | 8/-/47.1 | 8/-/42.9 | 8/-/42.9 | dysarthria |  | Yes | 10 | 14.7 | 0.9 | 262 |
|  |  | 43.2±9.8 | 33.4±12.0 | 9.8±8.4 |  |  |  |  |  |  |  |  |  |  |  |  |  | 10.6±0.5 | 14.8±0.7 | 2.2±0.9 | 328.5±142.2 |

Values are presented as the mean ± SD.

WCRS: Writer’s Cramp Rating Scale, TMDS: Tubiana’s Musician Dystonia Scale, ADDS: Arm Dystonia Disability Scale

※: Adverse events related to thalamotomy is shown. Detailed adverse events are shown in Supplementary table 2.

†: AP: anteroposterior from the posterior commissure, RL: right-to-left from the midline, SI: superoinferior to the anterior commissure-posterior commissure plane

‡: Lesion Volumes were measured on a thin-slice T2 weighted sequence.
