# Supplementary material for: Magnetic Resonance‐Guided Focused Ultrasound Thalamotomy for Focal Hand Dystonia: A Pilot Study
Source: Mov Disord. 2021 May 29;36(8):1955–9. doi: 10.1002/mds.28613 (PMC8453941; doi:10.1002/mds.28613)
Supplement: Supplementary file 4 — Table S2. Summary of focused ultrasound procedures [file MDS-36-1955-s005.docx]

Supplementary Table.2 Summary of focused ultrasound procedures

|  |  |  |  | Temperature | |  |  |
| --- | --- | --- | --- | --- | --- | --- | --- |
| Case | SDR | Number of sonications | Range of energy delivered (J) | max | averege | Number of soniactions above 55 degrees | Lesion Volume  (mm³) |
| 1 | 0.43 | 13 | 1500-26115 | 63 | 59 | 4 | 282 |
| 2 | 0.34 | 15 | 2495-31198 | 59 | 55 | 3 | 324 |
| 3 | 0.34 | 16 | 3000-31834 | 60 | 54 | 4 | 226 |
| 4 | 0.38 | 9 | 2485-31249 | 58 | 56 | 4 | 392 |
| 5 | 0.37 | 6 | 2485-14822 | 69 | 64 | 4 | 442 |
| 6 | 0.4 | 10 | 3000-37531 | 56 | 54 | 2 | 155 |
| 7 | 0.34 | 11 | 2500-36088 | 57 | 55 | 4 | 255 |
| 8 | 0.45 | 9 | 1514-29063 | 58 | 55 | 4 | 286 |
| 9 | 0.36 | 12 | 2000-32050 | 61 | 59 | 6 | 661 |
| 10 | 0.35 | 9 | 2004-31030 | 60 | 55 | 6 | 262 |
|  |  |  |  | 60.1±3.7 | 56.6±3.2 |  | 328.5±142.2 |

Plus-minus values are means ±SD

SDR: skull density ration
