# Supplementary material for: Magnetic Resonance‐Guided Focused Ultrasound Thalamotomy for Focal Hand Dystonia: A Pilot Study
Source: Mov Disord. 2021 May 29;36(8):1955–9. doi: 10.1002/mds.28613 (PMC8453941; doi:10.1002/mds.28613)
Supplement: Supplementary file 5 — Table S3. Adverse events [file MDS-36-1955-s002.docx]

Supplementary Table.2 Adverse events

|  | |  | **Transient** | **12 months** | - |
| --- | --- | --- | --- | --- | --- |
| **Related to thalamotomy** | | |  |  |  |
| Dysarthria | |  | 3 | 1 |  |
| Unsteady gait | | | 2 | 0 |  |
| Facial palsy | | | 2 | 0 |  |
|  |  | |  |  |  |
| **Related to stereotactic frame** | | |  |  |  |
| Headache | |  | 10 | 0 |  |
| Occipital sensory loss | | | 3 | 0 |  |
| Periorbital edema | | | 1 | 0 |  |
|  |  | |  |  |  |
| **Related to sonication** | | |  |  |  |
| Headache | |  | 10 | 0 |  |
| Nausea | |  | 3 | 0 |  |
|  | |  |  |  |  |
| **Unlikely to be related** | |  |  |  |  |
| Suicide attempt | | |  |  | 1 |

Date are number of patients
